# Vertebral Body Tethering: Indications, Surgical Technique, and a Systematic Review of Published Results

**DOI:** 10.3390/jcm11092576

**Published:** 2022-05-04

**Authors:** Arimatias Raitio, Johanna Syvänen, Ilkka Helenius

**Affiliations:** 1 Department of Paediatric Surgery, Turku University Hospital, University of Turku, Kiinamyllynkatu 4-8, 20521 Turku, Finland; arimatias.raitio@fimnet.fi; 2 Department of Paediatric Orthopaedics, Turku University Hospital, University of Turku, Kiinamyllynkatu 4-8, 20521 Turku, Finland; johanna.syvanen@tyks.fi; 3 Department of Orthopaedics and Traumatology, Helsinki University Hospital, University of Helsinki, Topeliuksenkatu 5, 00260 Helsinki, Finland

**Keywords:** adolescent idiopathic scoliosis, growth-friendly techniques, surgery, vertebral body tethering

## Abstract

Vertebral body tethering (VBT) represents a new surgical technique to correct idiopathic scoliosis using an anterior approach, spinal instrumentation with vertebral body screws, and a cable compressing the convexity of the curve. According to the Hueter-Volkmann principle, compression reduces and distraction increases growth on the growth plates. VBT was designed to modulate spinal growth of vertebral bodies and hence, the term ‘growth modulation’ has also been used. This review describes the indications and surgical technique of VBT. Further, a systematic review of published studies was conducted to critically evaluate the results and complications of this technique. In a total of 23 included studies on 843 patients, the preoperative main thoracic curve corrected from 49 to 23 degrees in a minimum 2 year follow-up. The complication rate of VBT was 18%. The results showed that 15% of VBT patients required reoperations for pulmonary or tether-related issues (10%) and less than 5% required conversion to spinal fusion. While the reported median-term results of VBT appear promising, long-term results of this technique are currently lacking.

## 1. Introduction

Adolescent idiopathic scoliosis (AIS) is a three-dimensional deformity including a lateral deviation of the spine, reduced thoracic kyphosis, and rotation of the vertebral bodies. A curve of 45 degrees or higher is typically regarded as an indication to surgical treatment as these curves typically continue to progress even in skeletally mature patients [1]. Additionally, thoracic curves of over 50 degrees are associated with reduced lung volumes [2]. 

Three-dimensional correction of scoliosis and continued growth should be the aim of the treatment of spinal deformity on a growing child [3]. Posterior spinal fusion with pedicle screw instrumentation has been the traditional method to address these curves [4]. Normal lung development is dependent on the length of the thoracic spine and its final length is closely related to the lung volume obtained at skeletal maturity [5]. A recommended minimum length of the thoracic spine before posterior fusion is 22 cm [6,7,8]. Additional length obtained from correction of spinal deformity averages about 25 mm in normal AIS [9].

Spinal fusion provides sustainable long-term outcomes but is associated with reduced spinal mobility [10] and hence reduced functional outcomes as compared with the normal population [11]. On the other hand, it leads to an irreversible stage of permanent spinal fusion and straining of the remaining mobile segment due to reduced spinal mobility [12,13]. These disadvantages have led surgeons to investigate other methods to correct adolescent idiopathic scoliosis without spinal fusion.

It is known that every human vertebral body between C3 and L5 has a growth plate (apophysis) on its upper and lower endplates. Both endochondral ossification (length) and appositional ossification (volume) lead to growth of the spine [14]. According to the Hueter-Volkman principle, distraction of the growth plate promotes and compression inhibits growth [15]. To control this growth, surgeons have attempted asymmetrical hemiepiphyseodesis to the spine, but this has remained unpredictable [14,16]. Asymmetrical growth plate inhibition with staples or unilateral plates has been used for decades in mechanical axis deviations (e.g., genu valgum or varum) of lower extremities in growing children [17]. A similar technique was applied by Betz and colleagues in the spine using stapling over the disc and growth plates. However, this was only successful in thoracic curves less than 35 degrees, which are typically treated with a brace on a growing child [18]. Additionally, movement of the spine often led to problematic loosening of the vertebral implants extending over the intervertebral disc [19].

Spinal tethering is the newest method to address scoliosis deformity correction three-dimensionally without fusion in preadolescent patients. It is based on the Hueter-Volkman principle and utilizes the patient’s own spinal growth to improve the initial correction rate after surgery. Currently, spinal tethering is mainly indicated only in children with suitable growth remaining (Sanders 2 to 5), while spinal arthrodesis can be performed whenever a minimum of 22 cm of the thoracic spine length has been achieved. Additionally, spinal tethering maintains spinal mobility and can be converted to anterior or posterior spinal fusion if necessary. A systematic review of the literature was conducted to analyze the published results and complications of VBT. Additionally, indications and technical considerations of this technique are described.

## 2. Methods

### 2.1. Identification and Selection of Studies

A comprehensive search of the published literature in PubMed and EMBASE databases was performed based on PRISMA (Preferred Reporting Items for Systematic Reviews and Meta-Analyses) guidelines [20] using ‘vertebral body tethering’ as a keyword. 

### 2.2. Inclusion and Exclusion Criteria

All articles reporting a minimum of one-year follow-up results of AVBT published up to February 28, 2022, were included. Non-English language papers, animal studies, and case reports (<3 patients) were excluded.

### 2.3. Data Extraction and Analysis

Identified papers were independently reviewed by two authors (AR and JS) with final selection approved by the senior author (IH). The data on patient demographics, pre- and postoperative scoliosis curves, duration of surgery, intraoperative blood loss, length of follow-up, and complications were extracted from the original publications.

### 2.4. Statistical Analysis

Analyses were performed using JMP Pro, version 16.1.0 for Windows (SAS Institute Inc., Cary, NC, USA).

## 3. Anterior Vertebral Body Tethering

Lately, spinal tethering has become one of the options to treat AIS without spinal fusion. This method has been made possible with better understanding of spinal biomechanics, technical developments in minimally invasive techniques (endoscopic and mini-open), and improved instrument and device design [21,22,23]. The tethering system limits the progression of scoliotic deformity by mechanically restraining the remaining spinal growth internally [19].

**Figure 1:**
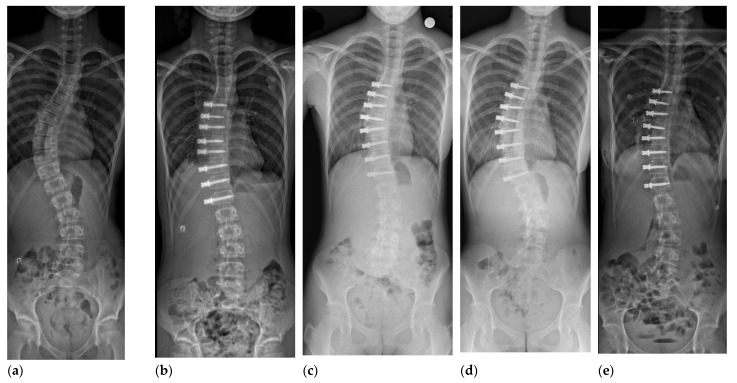
12 year-old girl, Sanders 2, Lenke 1 AN curve of 50 degrees (**a**). First erect postoperative radiograph (**b**), 1 year follow-up (**c**), 30 month follow-up demonstrating splaying of the screw heads at multiple levels and progression of the deformity (**d**), and after revision surgery (Sanders 6) for replacement of broken tether (**e**).

Spinal tethering is carried out using an anterior thoracoscopic or mini-open thoracolumbar approach. One or two bicortical screws over a washer or a small plate are inserted to each vertebral body laterally. A polyethylene tetraphalate cable is used to connect these screws. Immediate correction of the deformity is obtained by compressing the cable and between the screws (typically 45–50% initial correction). Additional correction can be obtained via growth modulation of the vertebral bodies according to the Hueter-Volkmann principle (Figure 1).

### 3.1. Indications for Main Thoracic Curves

The most well-documented indication for spinal tethering is a single major thoracic curve with non-structural lumbar and proximal thoracic curve (Lenke 1A or 1B curve in a preadolescent patient [19,23,24]. Recently, Krakow et al. [25] evaluated how many AIS patients would potentially be suitable candidates for VBT. In their study, approximately 25% of the patients fulfilled the growth parameters and curve characteristics (Lenke 1, 3, 5, or 6 curves, i.e., not including a structural upper thoracic curve) amenable to VBT. Therefore, the majority of scoliosis patients may still require posterior spinal fusion (PSF).

Skeletal growth can be assessed using the hand radiograph and Sanders’ classification [26]. Patients with a relatively flexible right thoracic curve (40 to 60 degrees, bending to 30 degrees or below), rib hump of less than 20 degrees, and suitable amount of remaining growth (Sanders 3 to 4) are the ideal candidates for the tethering procedure [19,24].

### 3.2. Timing of the Procedure

Appropriate timing of VBT is of utmost importance. If carried out too early, the patient may undergo overcorrection (i.e., right-sided curve turns into left-sided curve). Additionally, the remaining growth modulation may not correct the curve enough and/or a tethering rupture may result if the procedure is performed too late. In cases with limited growth remaining, or no growth at all, immediate correction utilizing the mobility of the discs can be achieved intraoperatively. However, this correction may not be maintainable without substantial three-dimensional shape change of the vertebral bodies via growth [19]. Anterior shortening may help with the restoration of thoracic kyphosis, but according to the literature, this kyphosing effect seems to be minimal [27].

Alanay et al. [28] investigated the effects of skeletal maturity according to Sanders’ classification (hand radiograph) on postoperative growth modulation. They observed that growth modulation was unpredictable in Sanders 1 (prepubertal) resulting in up to 45 degrees and in Sanders 2 (start of puberty) resulting in up to 29 degrees of postoperative growth modulation. According to their findings, Sanders 3–5 were the most predictable in terms of growth modulation of VBT.

In the study of Takahashi et al. [24], the average correction rate of thoracic segments was 1.8 per segment per year for the first 2 years. Significantly greater rates were observed for the Sanders stage two than the Sanders stage three cohort. In the same study, scoliosis correction correlated also with height velocity. Still, there is a lack of studies to determine the optimum timing of the procedure and the optimum amount of tension that should be placed.

### 3.3. Technical Considerations for Vertebral Body Tethering

The procedure is carried out using a strict lateral decubitus position and single lung ventilation. Instrumentation is typically carried from end vertebra to end vertebra. The spine is accessed anteriorly with mini-open, open thoracotomy, or thoracoscopically [23]. To minimize the chest wall violation and associated deleterious effects on pulmonary function, most surgeons favor minimally invasive techniques [15]. Preoperative screw trajectory planning under C-arm fluoroscopy helps planning the portal placement. 

The right lung should be deflated during surgery. Parietal pleura is opened over the spine using a monopolar hook or ultrasonic sealing device such as Harmonic scalpel (Ethicon Endo-Surgery, Inc, Cincinnati, OH, USA). Segmental vessels are ligated or mobilized on the convex side. Fluoroscopic control is used to control the placement of staples and bicortical screws. A polyethylene tether is placed and tightened starting cranially. Tightening can be controlled using a force measurement. Typically, the apical segments are tightened into 300–400 Newtons and upper thoracic screws to maximum 150–200 N to prevent screw pull-out. A chest drain is typically placed and set into 10–20 cm H_2_O suction.

Endoscopic vertebral body tethering involves a relatively long learning curve [29]. Reported operation time for AVBT ranges from 2.7 to 4.3 h with a mean of 3.8 h in this systematic review [30,31]. Intraoperative blood loss is typically minimal averaging at 180 mL, while the length of stay ranges from 3 to 5 days postoperatively [29]. Screw accuracy can be improved using CT-guided navigation, or as we have adopted, intraoperative imaging using intraoperative 3D evaluation of the screws and staples inserted before corrective maneuvers. Especially in the upper thoracic spine, the vertebral bodies are small with limited margins around the implants.

If the mini-open technique with a small thoracotomy is used, the segmental vessels can be mobilized especially in the apical area, while with the thoracoscopic technique, all the segmental vessels need to be ligated. The mini-open technique also allows easier spinal manipulation in terms of derotation and tightening of the cord. On the other hand, the thoracoscopic technique might be associated with reduced postoperative pain, better cosmesis, and better pulmonary function due to less chest wall violation. However, there are no studies comparing these two approaches. It should be noted that in revision cases, spared segmental vessels might start to bleed profusely as the cable on top of them is firmly attached to them.

### 3.4. Indications for Thoracolumbar and Lumbar Curves

Growth modulating techniques are not contraindicated in lumbar curves. However, caution needs to be taken as most techniques have been described for thoracic curves [32]. Thoracolumbar or lumbar idiopathic scoliosis (Lenke 5 type curve) may be an option for spinal tethering, as loss of spinal mobility in this area has an even greater impact on functional outcomes. However, there are few studies on this indication [33,34]. Approach includes a mini-open thoracoabdominal exposure with two incisions: one over the 10th rib and a second over the L3/4 disc. Lumbar vertebral bodies have larger diameters and the use of two screws and two cables is easier in this area. When two curves (thoracic and lumbar) are instrumented, T12 typically needs instrumentation from right (thoracic curve) and left (thoracolumbar) sides. Careful evaluation of the sagittal profile reduces the risk of flat back or decreased lordosis [32].

## 4. Results

**Figure 2:**
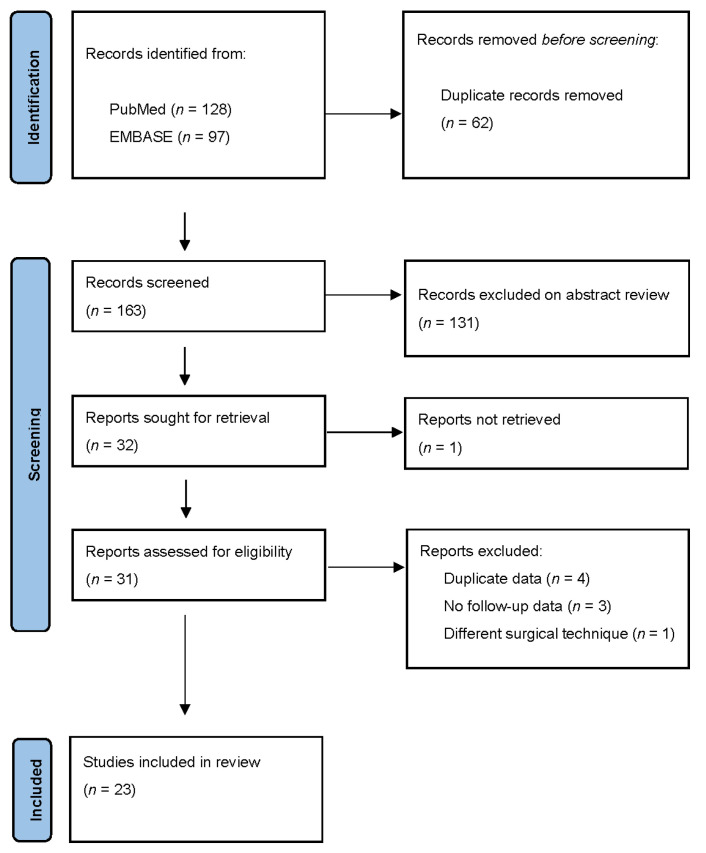
PRISMA study selection flow diagram.

Our literature search identified 163 publications after duplicates were excluded. Thirty-one papers met inclusion criteria and were selected for full text review (Table 1). After full text review of 31 articles, 23 papers met the eligibility criteria and were selected for review (Figure 2). A total of 843 patients (736, 87% women) with a mean age of 12.7 years underwent VBT and were followed-up for minimum of 2 years.

### 4.1. Curve Correction after AVBT in Thoracic Curves

In the included studies, the mean preoperative main thoracic curve was 49 degrees, which corrected to 24 degrees in first postoperative imaging. VBT provided sustainable median-term results as the reported curves after a minimum of two-year follow-up averaged at 23 degrees. Kyphosis remained unchanged at 23 degrees. Samdani et al. [22] observed that the lumbar curves underwent spontaneous correction from 25 degrees to 7 degrees in two years. In addition, axial rotation measured by scoliometer improved from 12 degrees to 7 degrees at the latest follow-up in their cohort. 

Newton and coworkers recently published a follow-up study of 14 AVBT patients using biplanar radiographs (EOS) [50]. In their 3D models, seven patients (50%) showed progressive correction of scoliosis defined as ≥15 degrees scoliosis correction between postoperative and follow-up radiographs. Coronal vertebral wedging occurred at 0.11°/month in the progressive correction compared to 0.02°/month in the non-progressive group. Similarly, coronal disc wedging was more pronounced in the progressive than in the non-progressive group. They concluded that the symmetry of apical vertebrae and the height of the discs in immature patients with thoracic scoliosis could be restored. Progressive correction was dependent on the skeletal maturity. According to Takahashi et al. [24], twice as much correction occurred in the Sanders stage 2 compared to the Sanders stage 3 group.

### 4.2. Outcomes of Lumbar Curves and Double Curves

Compared to thoracic scoliosis, there are limited studies on the correction of lumbar and double curves using VBT. A single lumbar tether seems to have a relatively high cord breakage up to 50% within two years [36,51]. Limited evidence suggests that using a double tether with double screws can reduce this risk to 16% during the first year [33]. Pehlivanoglu et al. [34] reported the outcomes of 13 patients (11.8 years at the time of surgery) undergoing both endoscopic tethering of the thoracic curves (mean preoperative curve of 48 degrees) and mini-open approaches for thoracolumbar and lumbar curves (mean curve 45 degrees). They observed an initial 64% correction of thoracic and 69% of lumbar curves with additional growth modulation resulting in 80% and 82% correction at 2 year follow-up, respectively.

### 4.3. Reported Complications

The reported rate and nature of complications for AVBT appear acceptable. In the included studies, the complication rate was 18% with pulmonary (pneumothorax, pleural effusion) and instrumentation-related (tether breakage, overcorrection) being the most common. Reoperations related to tethering were required in 10% of cases. These included tether release(s) for overcorrection, replacing and extending tethers for breakage or curve progression, and chest tube insertions for pulmonary complications. The vast majority avoided spinal fusion, as only 4.7% of VBT patients required conversion to PSF after unsuccessful tethering.

However, the published studies on outcomes and complications of AVBT are sparse. Hence, the reported rate of complications varies considerably between reports and the true complication rate remains to be established. Furthermore, long-term studies related to complication and reoperation rates are lacking.

### 4.4. Comparison between Spinal Fusion and Vertebral Body Tethering

There was only one study comparing traditional fusion and AVBT. Newton et al. [44] compared the outcomes of AVBT and PSF using pedicle screw instrumentation at a mean of 3.5 years follow-up. The correction of major thoracic curves was significantly better in the PSF group (70%) as compared with AVBT (38%). There were nine revisions in the AVBT group including three conversions into PSF with three more pending. Twelve patients had a broken tether, but the majority (74%) of the patients in the AVBT cohort had avoided spinal fusion at the end of follow-up. Operative time was reported to be significantly shorter in AVBT than PSF while there was no difference in the length of postoperative stay [44].

Compared to AVBT, posterior spinal fusion is a permanent stage which cannot be reversed. The risk of revision after PSF remains low and is mainly related to deep surgical site infection, adding-on phenomenon, and rarely on pseudoarthrosis. The revision risk after VBT appears acceptable in the light of these comparisons given that PSF with pedicle screws is doable with small modifications and probably with similar outcomes than in primary surgery.

### 4.5. Spinal Mobility after AVBT 

Only a handful of studies have investigated spinal mobility after AVBT. Buyuk et al. [39] investigated the spinal mobility using flexion-extension and side bending radiographs in 32 children after thoracic VBT. These patients maintained both coronal (mean 7 degrees) and sagittal arc of motion (21 degrees) at one-year follow-up even though especially the coronal movement was significantly reduced from a preoperative value of 30 degrees. Another recent study demonstrated that AVBT in thoracolumbar curves yielded significantly superior lumbar range of motion and lumbar anterior and lateral flexibility compared to patients with spinal fusion. In addition, trunk flexor-extensor endurance and trunk motor strength were better in AVBT than PSF [34].

### 4.6. Pulmonary Function after AVBT

Baroncini et al. [52] evaluated the pulmonary function after mini-open VBT for AIS. Fifty-one patients completed pulmonary function testing including total lung capacity (TLC), forced vital capacity (FVC), and forced expiratory volume in one second (FEV1). There was a small reduction in FVC from 91% preoperatively to 86% at one-year follow-up, while TLC and FEV1 remained at the same level. They concluded that the mini-open approach does not result in a clinically significant reduction in pulmonary function. Further, Alanay et al. [53] and Samdani et al. [47] have reported significantly improved pulmonary function after VBT scoliosis correction.

### 4.7. Health-Related Quality of Life

Newton et al. [44] and Qiu et al. [54] reported similar HRQoL total and domain scores between AVBT and PSF patients. On the other hand, HRQoL and patient satisfaction were also significantly better in tethered patients in the study of Pehlivanoglu et al. [34]. Further, Hegde et al. [30] reported significant improvement in SRS-22 scores from preoperation to 1 year after surgery.

### 4.8. Cost-Utility Analysis

Only one study was found in the literature concerning costs of two different treatments [55]. It suggested that AVBT may be a cost-effective alternative to fusion. The results relied on HRQoL benefits over fusion patients.

## 5. Discussion

The premise of spinal growth modulation is supported by the basic science and experimental studies, which have shown that asymmetric mechanical compression of vertebral body centers can slow the growth on the anterior and convex aspect of the spinal column [15,56,57,58,59,60]. Similarly, clinical experience and publications of the early outcomes have confirmed the reduction in scoliosis curves over time with growth [19] as also noted in our systematic review.

As stated above, AVBT produces three-dimensional deformity correction during surgery which continues based on the Hueter-Volkman principle, producing asymmetrical growth to vertebras [61]. The same kind of technique can also be used from the posterior approach (costo-vertebral). However, the anterior technique has been proven to be more effective in all planes [62] in a finite element model. The anteriorly placed tether developed coronal correction, reduced axial rotation, and maintained kyphosis. In the same study, higher initial tensions produced overcorrection to the deformity. In another finite element model, tensioning of the cable 100 N vs. 200 N, and placing the screws on the lateral sides of the vertebral bodies (lateral, anterior, or triangulated) were important factors for ideal correction. That study demonstrated that a 200 N tightening and an anterior location provided better correction rates in all three planes [63]. Overall, the AVBT appears to be an effective technique in these models. 

### 5.1. Advantages

The main advantage of AVBT is allowing correction of the scoliotic deformity without reverting to spinal fusion, which could be avoided in the majority of patients according to this review. Initial correction is achieved with implants inserted thoracoscopically or through (mini-open) thoracotomy. Further correction is gained through the axial growth modulated by the inserted tether. Ideally, it has the potential to correct all three planes of deformity: compression of the apex in coronal plane via growth inhibition, correction of hypokyphosis by anteriorly placed screws and applied compression, and correction of axial rotation as these modulating forces are applied laterally.

AVBT is also less invasive than PSF. Hence, a more rapid return to normal daily life and sports can be expected, especially if a minimally invasive technique is applied [64]. Further, thoracoscopic operation is likely to be associated with minimal respiratory issues as well as minimal blood loss [65]. Additionally, avoiding spinal fusion provides the advantage of preserving at least some extent of spinal column mobility.

### 5.2. Disadvantages

The most common adverse events of AVBT consist of those related to thoracic surgery in general such as atelectasis and pneumo-, hemo-, and chylothorax, the need to convert endoscopic to open approach, and post-thoracotomy pain. Other adverse events include overcorrection, screw pull-out, and broken tether; all of which may require reoperation. Additionally, there are few published reports on the clinical outcomes of AVBT, and long-term outcomes remain elusive. Currently, only a small number of teams have published their data and the results remain to be substantiated by third parties. According to our systematic analysis, the rate of these adverse outcomes was 18%.

Theoretical disadvantages include concerns on the long-term sustainability of the results. Currently, we are unable to predict the fate of the tethered intervertebral discs and the effects of AVBT on the development and growth of the spinal canal. 

Contrary to spinal fusion and brace treatment, we do not yet know the long-term outcomes of AVBT. Pekmezci et al. [66] observed a reduction in the enlargement of the spinal canal by growth on a porcine model of anterior fusion. Even though this was not demonstrated in a tethering model, there is a chance that patients may end up with stiff degenerated thoracic spines associated with spinal stenosis in these segments after AVBT. Recently, Hoernschemeyer et al. [67] reassuringly reported that AVBT did not produce degenerative changes in the intervertebral discs or facet joint during two-year follow-up.

### 5.3. Limitations

There were no randomized controlled clinical trials or even prospective follow-up studies comparing the outcomes of AVBT and segmental pedicle screw instrumentation. Thus, we are currently lacking evidence-based recommendations on which to treat patients with instrumented spinal fusion and to use AVBT. Furthermore, long-term outcomes of AVBT are currently lacking.

## 6. Conclusions

Treatment of deformities in growing children is complex. Until recently, the surgical treatment has led into spinal fusion surgery. As a relatively novel technique, vertebral body tethering allows correction of the scoliotic deformity while preserving motion especially in patients with moderate curvature. The majority of these patients seemed to avoid posterior spinal fusion with a major curve less than 35 degrees, when followed-up to skeletal maturity. However, some of these patients required replacement of a broken tether, and overall risk of revision surgery appears to be around 15%. Additionally, long-term studies are required to clarify curve characteristics, rate of complications, and their prevention. 

## Figures and Tables

**Table 1:** Summary of eligible studies and their main findings. Values are given as mean (range) unless mentioned otherwise.

Author/Setting/Year	Number of Patients (% Women)	Age (Years)	Preoperative Curve	Final Curve	Length of Follow-Up (Years)	Complications (%)	Main Findings/Conclusion
Abdullah [35]/Multi-center register study/2021	120 (84.2%)	12.6 (8.2–15.7)	51.2 (40–70)	27.5 (−5–52)	2	15.8	Higher than expected complication rate during learning curve.
Alanay [28]/Single-center/2020	42 (95.2)	12.1 (SD 1.5)	47 (35–68)	17 (−6–28)	2.8	7.1	Curve behavior after VBT varied according to Sanders stage.
Baker [36]/Single-center/2021	17 (70.6)	12.9 (SD 1.4)	45 (35–60)	20 (−40–25)	2	23.5	The majority of patients (53%) were successful despite four revisions and nine broken tethers.
Baroncini [31]/2 centers/2021	86 (83.7)	13.2 (SD 2.4)	52.4 (SD 13.9)	26.6 (SD 12.7)	2	8.1	The majority of the patients had a physiologic sagittal profile after surgery.
Bernard [37]/Single center/2022	20 (95.0)	13.8 (9–17)	56.5 (40–79)	19.4 (−17–56)	5.4	15	High success rate (95%) in helping children avoid fusion at five years post-surgery.
Betz [38]/Single center/2019	71 (83.1)	14.5	N/A	N/A	2	4.2	Results of showed clinical success in 93% of immature patients, 81% of maturing, and 86% of mature patients.
Buyuk [39]/ Single center/2021	32 (93.8)	13 (11–15)	51 (42–70)	26 (7–43)	1	9.4	Particularly, sagittal plane motion was preserved postoperatively after anterior vertebral body tethering.
Cebeci [40]/ Single center/2017	12 (100)	12.2 (11–13)	46 (35–59)	18 (6–26)	2	0	VBT resulted in a significant correction in both major and compensatory curves.
Costanzo [41]/Single center/2022	23 (82.6)	12 (9–14)	56.5 (33–79)	37 (15–58)	2	8.7	Initial results were encouraging.
Hegde [30]/Single center/2021	10 (100)	14.9 (12–17)	52 (42–80)	15.3 (3–28)	2	0	Preliminary experience was promising.
Mackey [42]/Multicenter/2022	37 (97.3)	11.3 (IQR 10.9–11.8)	50 (IQR 43.5–58)	28 (IQR 21–35)	3	27	Satisfactory curve control and improved thoracic and spinal height.
Miyanji [27]/Multicenter/2020	57 (94.7)	12.7 (8.2–16.7)	51 (31–81)	23 (−18–57)	3.4	28.1	Satisfactory curve correction and an acceptable complication rate in skeletally immature patients.
Mladenov [43]/Single center/2021	20 (70.0)	13.4 (11.5–14.5)	46.5 (29–64)	23 (8–38)	1.6	5	Anticipated curve correction averaged 50%.
Newton [44]/Single center/2020	23 (69.6)	12 (9–15)	53 (41–67)	33 (−5–62)	3.4	39.1	AVBT resulted in less deformity correction and more revision procedures than PSF, but resulted in the delay or prevention of PSF in the majority of patients.
Pehlivanoglu [45]/Single center/2020	21 (71.4)	11.1 (9–14)	48.2 (IQR 44–52.1)	10.1 (IQR 7.7–11.2)	2.3	9.5	AVBT was a safe and effective option in skeletally immature patients with AIS.
Rushton [46]/2 centers/2021	112 (92.9)	12.7 (8.2–16.7)	50.8 (31–81)	25.7 (−32–58)	3.1	22	Satisfactory deformity correction in majority of cases.
Samdani [47]/Single center / 2021	57 (86.0)	12.4 (10.1–15.0)	40.4 (SD 6.8)	18.7 (SD 13.4)	4.6	12.3	Our current study suggested VBT as a viable option for skeletally immature children with scoliosis.
Takahashi [24]/ Single center / 2021	23 (69.6)	12.2 (SD 1.6)	53 (SD 8)	N/A	3.4	30.4	Correction occurred primarily within 2 to 3 years after surgery.
Wong [48]/Single center/2019	5 (100)	12 (9–12)	40.1 (37.2–44.0)	25 (−12.4–58)	4	40	Of all patients, 60% avoided spinal fusion.
Yucekul [49]/Single center/2021	28 (82.1)	12.2 (10–14)	46 (SD 7.7)	12 (SD 11.5)	3.2	28.6	Intermediate discs and facet joints were preserved after growth modulation with VBT surgery.

IQR—interquartile range, N/A—not available, and SD—standard deviation.
